# Self-Assembled Rough Endoplasmic Reticulum-Like Proto-Organelles

**DOI:** 10.1016/j.isci.2018.09.020

**Published:** 2018-09-27

**Authors:** Qingchuan Li, Xiaojun Han

**Affiliations:** 1 State Key Laboratory of Urban Water Resource and Environment, School of Chemistry and Chemical Engineering, Harbin Institute of Technology, 92 West Da-Zhi Street, Harbin 150001, China

**Keywords:** Biomaterials, Materials Science, Molecular Self-Assembly

## Abstract

Nature has evolved elaborate, dynamic organelle morphologies for optimal organelle functions. Among them, cisternae stacks are the universal structure for most organelles. However, compared with the well-studied spherical cell/organelle membrane mimic, the fabrication of the ubiquitously present cisternal organelle-like membrane structures for organelle mimic remains a challenging task. Herein, rough endoplasmic reticulum (RER)-like helicoidal cisternae stacks were assembled to mimic the enzyme crowded environment in spatially confined RER cisternae. RER-like single helicoid, multiple helicoids, and secondary helix are all observed. Membrane electrostatics drives their formation and controls the percentages, which indicates the possible role of membrane electrostatics in RER shaping. The organelle-like cisternae stacks can reversibly expand and compress, which provides modulated crowded or de-crowded enzyme environment for biochemical reactions. This work provides advanced membrane models, and novel mechanisms for organelle shaping and helicoids formation, and holds great potential in biomimetics, cell biology, and advanced materials design.

## Highlights

• Rough endoplasmic reticulum-like helicoids were formed from phospholipids
• Membrane electrostatics controls helicoids formation and probably organelle shaping
• The helicoids reversibly expand and compress to control loaded molecule concentration
• A “breathing” modulated proto-organelle model is proposed based on the helicoids

## Graphical Abstract

**Figure:**
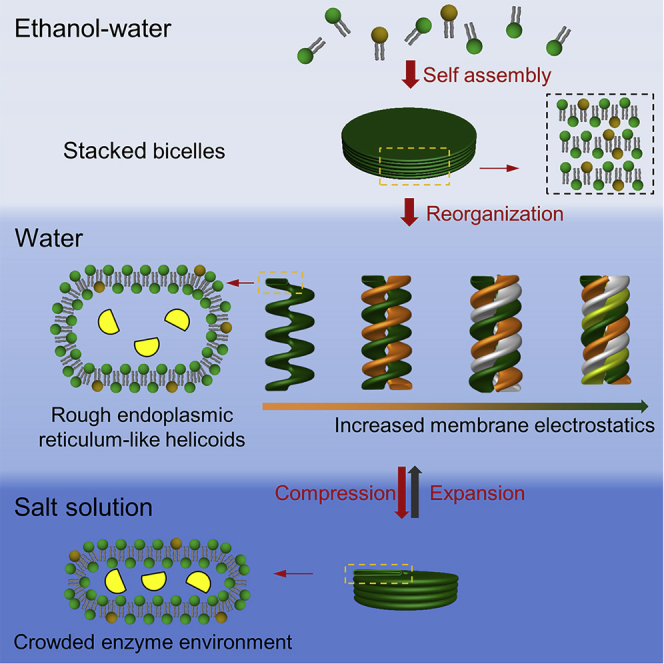

## Introduction

The existence of diverse intracellular membrane systems with characteristic morphologies was one of the most fascinating features of eukaryotic cells. Among them, cisternae stacks extensively found in Golgi apparatus, grana, and rough endoplasmic reticulum (RER) constituted the major component of intracellular membrane systems. However, although they shared similar cisternae-stacks-based morphologies, these intracellular membrane systems were essentially different in their spatial organization. The cisternae stacks in Golgi apparatus stacked close to each other via protein connection at a typical intercisternal distance of 7–15 nm (Tachikawa and Mochizuki, 2017, Mollenhauer and Morré, 1991). The grana existed as cylindrical stacks of flattened and closely appressed cisternae, which were interconnected by the fret-like stromal lamellae around the cylinder (Ruban and Johnson, 2015, Shimoni et al., 2005). On the contrary, the RER was recently discovered to be composed of a structural motif of helicoidal cisternae stacks resembling spiral stairs to form continuous membrane system (Terasaki et al., 2013). The mechanisms for the generation and maintenance of these different cisternae-stacks-based intracellular membrane systems are still less understood.

Artificial membranes provided simplified models for studying and emulating biological membrane systems. In the past decades, scientists have developed a variety of model systems, including vesicles (Deshpande et al., 2016), tubes (Bi et al., 2014), supported or suspended lipid bilayers (Tayebi et al., 2012, Osaki and Takeuchi, 2017, Fuhs et al., 2018), bicelles (Dürr et al., 2012), and nanodisks (Ravula et al., 2017). To further mimic the complexity of natural membrane systems, more complex membrane structures including multilamellar (Matosevic and Paegel, 2013) and multicompartmented vesicles (Zong et al., 2017, Deng et al., 2017), stacked bicelles or nanodisks (Matsui et al., 2015, Yang et al., 2014, Wang et al., 2018), and tube networks/stacks (Karlsson et al., 2001, Powers et al., 2017, Zhang et al., 2017) were recently developed. These artificial membrane systems supplied platforms for the study of protein structures and functions (Dürr et al., 2012, Zhang et al., 2016, Burré et al., 2010), the investigation of cellular processes (Fenz et al., 2017, Kamiya et al., 2016, Küchler et al., 2016), and biomimetics (Li and Han, 2018, Altamura et al., 2017, Adamala et al., 2016, Heath et al., 2017, Park et al., 2018), and provided important clues for membrane shaping (Powers et al., 2017, Shi and Baumgart, 2015). However, cisternae stacks, especially the helicoidal structures found in RER, were rarely artificially fabricated from phospholipids.

Herein, stacked bicelles incorporated with charged lipids were assembled in ethanol-water solution via a recrystallization process. When suspended in water, they quickly transformed to RER-like helicoidal cisternae stacks, which can further spiral to secondary helical structures. The influences of the parameters for stacked bicelles formation and reorganization were investigated to reveal the mechanisms for the formation of helicoidal cisternae stacks. Biomolecules such as fluorescent polymers or enzymes in the cisternae stacks can be reversibly concentrated and diluted for crowding of molecules. The formation of helicoidal cisternae stacks with encapsulated biomolecules provided RER-like proto-organelle models to study or mimic their functions and clues for the mechanisms of helicoidal structure formation in cells.

## Results and Discussion

### Cisternae Stacks Formation Composed of Continuous Membrane Systems

**Figure 1:**
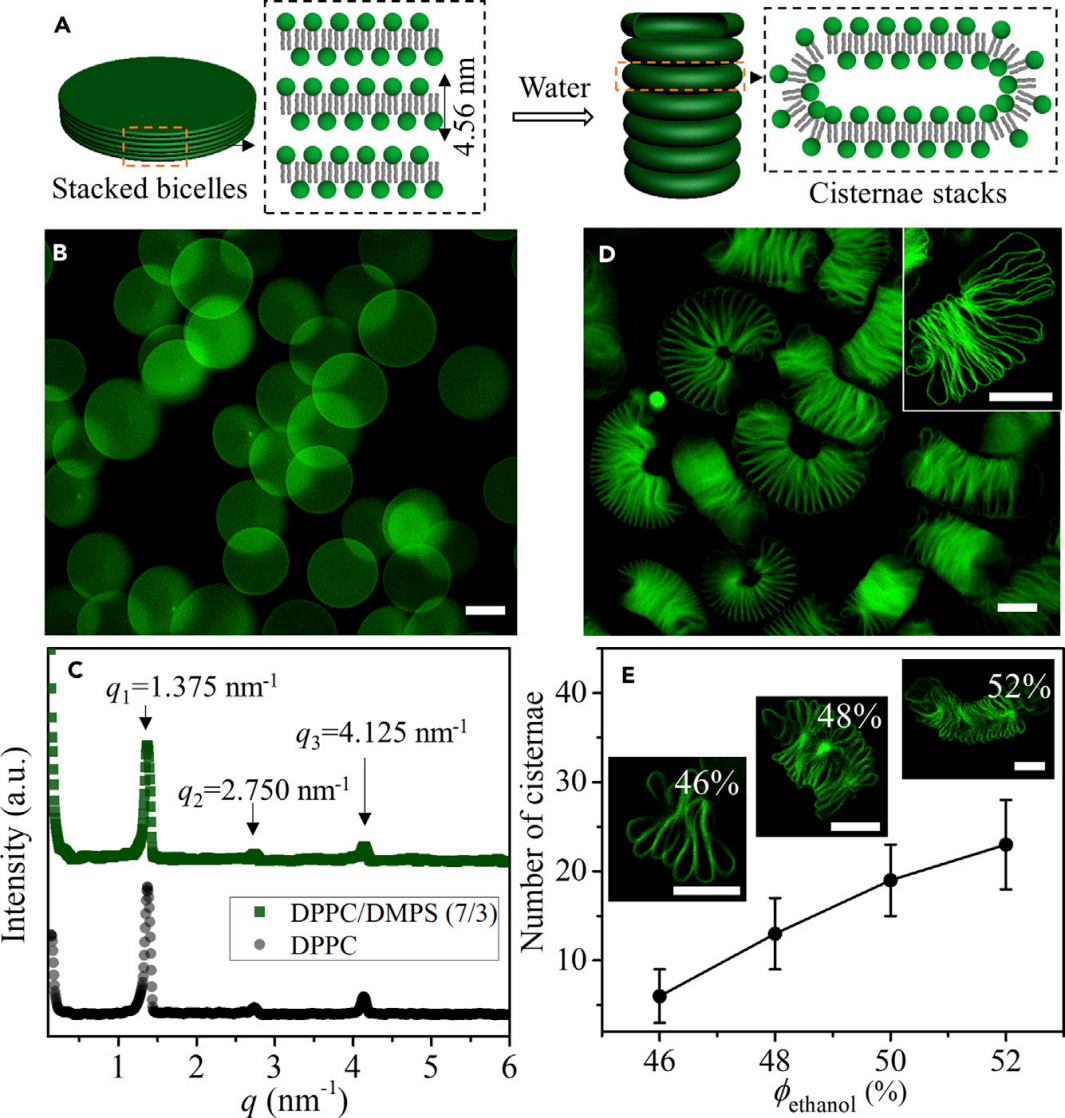
Cisternae Stacks Formation from Micro-Sized Stacked Bicelles (A) Schematic illustration of the transformation of stacked bicelles to cisternae stacks in water. (B) Fluorescence image of stacked bicelles formed in 50% ethanol solution. (C) SAXS profiles of stacked bicelles formed in 50% ethanol solution with different phospholipid compositions. (D) Fluorescence image of cisternae stacks transformed from stacked bicelles formed in 50% ethanol solution. The inset is the confocal fluorescence image of one cisternae stack. (E) Variation of the number of cisternae in cisternae stacks with ethanol volume percentage *Φ*_ethanol_ (*N* = 200). The insets are typical confocal fluorescence images. Phospholipid concentration of 0.10 mg/mL was used in these experiments. Scale bars, 10 μm. Error bars are ± SEM. See also Figures S1–S6.

Micro-sized stacked bicelles were formed from negatively charged 1,2-dipalmitoyl-*sn*-glycero-3-phosphocholine (DPPC)/1,2-dimyristoyl-*sn*-glycero-3-phospho-L -serine (sodium salt) (DMPS) (*w*/*w*, 7/3) mixtures by slowly cooling the phospholipid dissolved in 50% ethanol-water solution from 50°C to 25°C at a rate of 0.5°C/min (Figures 1A–1C). Similar to the results from zwitterionic DPPC (Li and Han, 2018), stacked bicelles from DPPC/DMPS (*w*/*w*, 7/3) were formed in a well-defined round shape with an average diameter of ∼15 μm, as depicted by the fluorescence image (Figure 1B). The small-angle X-ray scattering (SAXS) profiles of the stacked bicelles displayed only one set of lamellar diffraction peaks, at *q*_1_ = 1.375 nm^−1^, *q*_2_ = 2.750 nm^−1^, and *q*_3_ = 4.125 nm^−1^ (Figure 1C), which confirmed the periodic lamellar structure of the stacked bicelles with periodic spacing *d* of 4.56 nm, as given by *d* = 2π/*q*_1_. The periodic spacing of 4.56 nm included the thickness of lipid membrane and the thickness of solvent layer, which indicated the interdigitated phase in the bicelles (Adachi et al., 1995). The incorporation of negatively charged DMPS displayed no influence on the periodic spacing from the SAXS profiles (Figure 1C), which might be due to the screening of the repulsive forces among lipid bilayers in the ethanol-water solvent condition with relative lower dielectric constant. However, the existence of DMPS slightly decreased the main phase transition temperature *T*_m_ according to the differential scanning calorimetry results (Figure S1), from 40.2°C for DPPC bicelles to 39.4°C for DPPC/DMPS bicelles.

When dispersed in water or ethanol-water solution with ethanol volume percentage *Φ*_ethanol_ below 20%, the increased edge energy and reanimated electrostatic repulsive energy drove the prompt transformation of stacked bicelles to cisternae stacks in seconds after a short delay (Figures S2 and S3). These cisternae stacks with cisternae piled one by one can be clearly recognized from the fluorescence images (Figures 1D and S3). Water-soluble fluorescent markers Rhodamine B isothiocyanate-Dextran (∼70 kDa) were entrapped in the cisternae stacks during the reorganization process (Figure S4), which indicated the separated phase of the cisternae stacks from outer solution. The number of cisternae in the cisternae stacks can be modulated from ∼5 to ∼20 by the variation of *Φ*_ethanol_ for stacked bicelles formation (Figures 1E and S5). With increased *Φ*_ethanol_, cisternae stacks with more cisternae formed. The percentage of negatively charged DMPS had no obvious influence on the number of cisternae in the cisternae stacks (Figure S6).

### Confirmation of the Formation of RER-Like Helicoids and Multiple Helicoids

**Figure 2:**
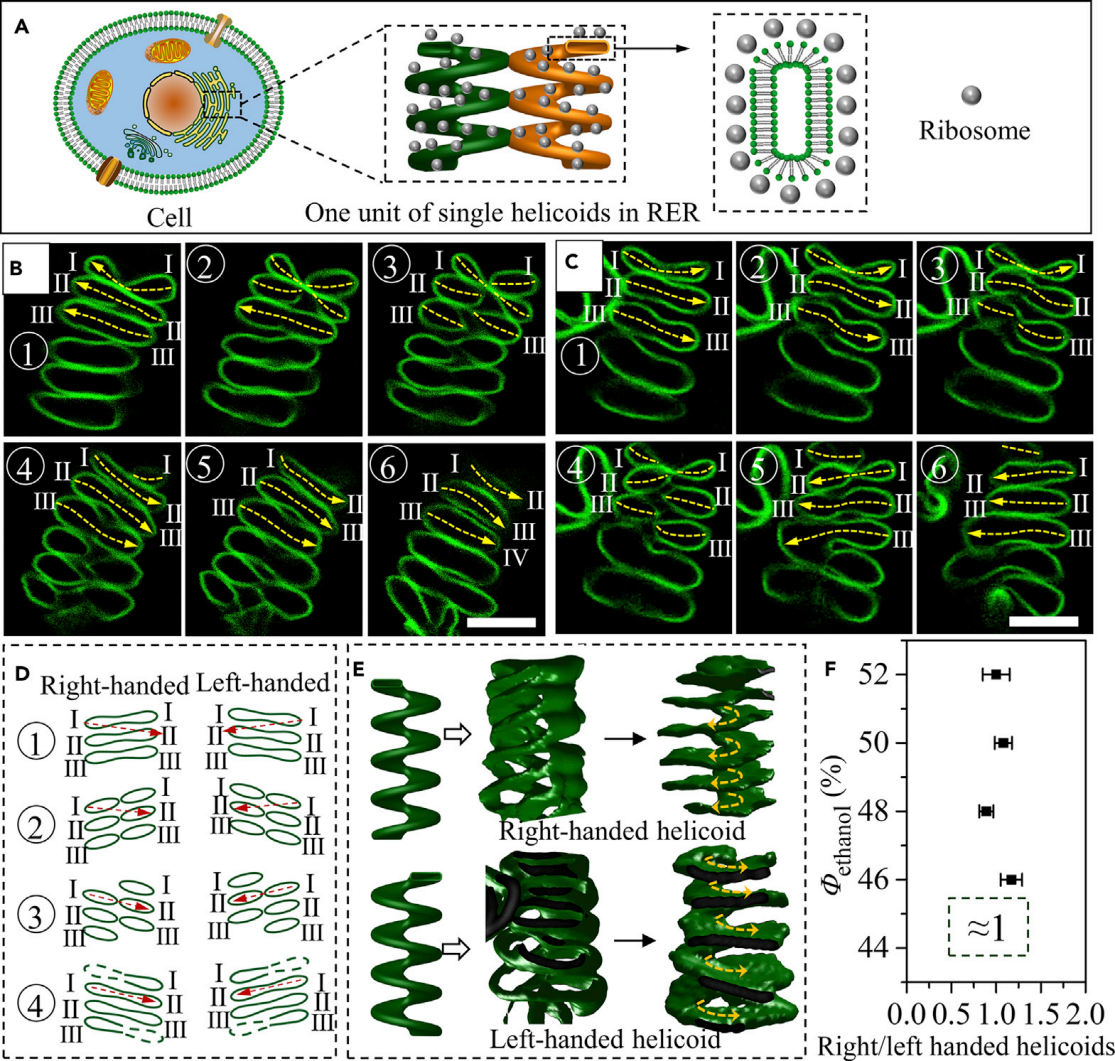
Formation of RER-Like Single Helicoidal Cisternae Stacks (A) Schematic illustration of RER in cells. (B and C) Typical fluorescence images of the right-handed (B) and left-handed (C) RER-like helicoids visualized in z -stacks by laser confocal microscopy. The yellow dash arrows indicate the midline of the cisternae. (D) Schematic illustration of variation of the cisternae in z stacks under laser confocal fluorescence microscopy for cisternae stacks with different chirality. The red dash arrows indicate that there exist connections between two cisternae. (E) The 3D reconstruction of the z stacks in (B) and (C) (middle) or the midline of the cisternae (right). The yellow dashed arrows indicate the spiral directions of the helicoids. (F) Counted ratio of right- to left-handed stacks formed in solution with different *Φ*_ethanol_. Scale bars, 10 μm. Error bars are ±SEM. See also Figure S7.

**Figure 3:**
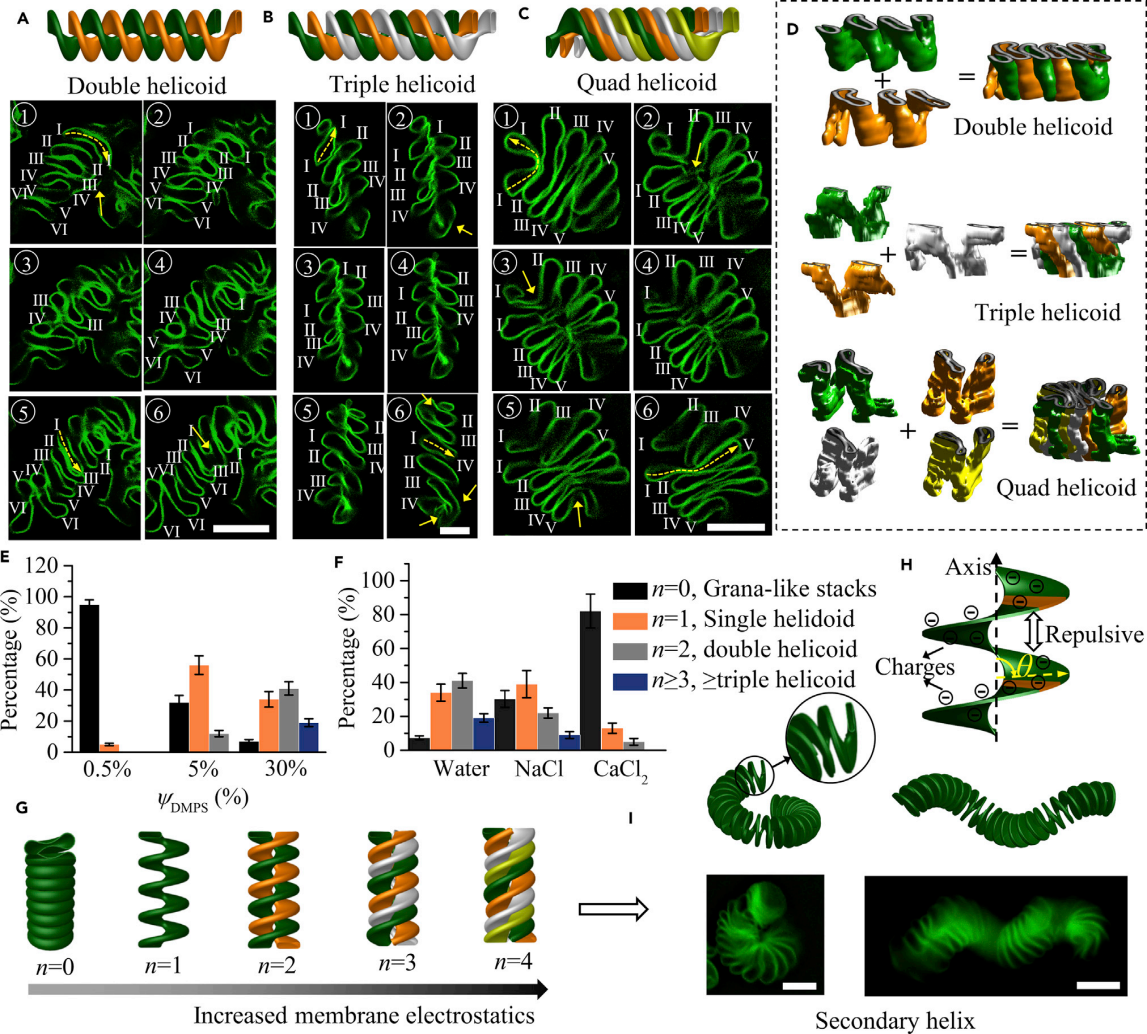
Formation of Multiple Helicoidal Cisternae Stacks and Secondary Helix (A–C) Schematic illustration and typical fluorescence images of the multiple helicoids visualized in z stacks of double (A), triple (B), and quad (C) helicoidal cisternae stacks. (D) 3D reconstruction of the multiple helicoidal cisternae stacks. Different helicoids in one cisternae stack were painted with different colors. (E) Influence of the percentage of negatively charged DMPS *ψ*_DMPS_ in DPPC/DMPS stacked bicelles on the percentages of cisternae stacks with different *n* (*N* = 100). (F) Variation of the percentages of helicoidal cisternae stacks (containing 30% DMPS) with different *n* when ions were added to screen the electrostatic repulsive forces during the reorganization process (*N* = 100). The stacked bicelles were formed in 50% ethanol solution and then dispersed into water to obtain cisternae stacks. (G) Schematic illustration of the influences of charged lipids on the formation of cisternae stacks with different *n*. (H) Schematic representation of a helicoid with angle of *θ* between the axis and local ramp plane. The orange surfaces indicated two parallel planes in the helicoid that repelled each other under electrostatic repulsive force. (I) The schematic and fluorescence images for the formation of secondary helix from the helicoidal cisternae stacks. Scale bars, 10 μm. Error bars are ±SEM. See also Figures S8–S19.

The cisternae stacks can maintain their morphology for at least 1 month at 25°C. However, when treated with heating or shaking, they paved different morphology evolution pathways (Figure S7), including spatially segmented multilamellar vesicles (Figure S7D), erythrocyte (Figure S7E). After heating at 35°C (below phase transition temperature *T*_m_ of 39.4°C) for about 12 hr, some of the cisternae stacks fully expanded and formed continuous membranes, such as randomly curved complete membranes or helix (Figure S7B), which indicated that the cisternae in the stacks might be physically connected to form continuous membrane systems. To identify how they were connected, fluorescence images of the cisternae stacks in z stacks at 0.32 μm were taken to construct the 3D structures of the cisternae stacks (Figure 2). Most of the cisternae stacks were proved to be in helicoidal structures with different number of helicoids *n* in one cisternae stack, including RER-like single helicoids (*n* = 1) (Figure 2) and multiple helicoids such as double (*n* = 2, Figure 3A), triple (*n* = 3, Figure 3B), quad (*n* = 4, Figure 3C), penta (*n* = 5, Figure S8), and hexa helicoids (*n* = 6, Figure S8). Single helicoidal cisternae stacks were previously reported to be the structural motif of RER in cells, where the right- and left-handed helicoids were alternatively connected to form continuous membrane systems (Figure 2A). In our work, both right- and left-handed helicoids were found. The confocal fluorescence images in z stacks (obtained from top to bottom) provided primitive evidence to quickly identify the chirality of the helicoids (Figures 2B–2D). As indicated by the yellow dashed arrows in Figures 2B and 2C and the schematic in Figure 2D, the cisternae in the stack were piled one by one with no obvious connections in panel 1. In subsequent panels (panels 2 and 3 in Figure 2B, panels 3 and 4 in Figure 2C, and panels 2 and 3 in Figure 2D), each cisterna disconnected into two parts. In the next set of panels, one part of the disconnected cisternae connected with the opposite part of neighboring cisternae depending on the chirality. For right-handed helicoids, the left part of a disconnected cisterna *s* tended to subsequently connect with the right part of another disconnected cisterna *s+*1. For example, the left part of cisternae Ⅰ, Ⅱ, and Ⅲ connected with the right part of cisternaeⅡ, Ⅲ, and IV, respectively, as indicated by the yellow dashed arrows in panel 6 in Figure 2B. On the contrary, for left-handed helicoids, the right part of cisterna *s* connected with left part of cisterna *s*+1 (panel 6 in Figure 2C and panel 4 in Figure 2D). The 3D reconstruction of the cisternae stack (middle in Figure 2E and Video S1) illustrated that the cisternae stack possessed twisted surface that has a handedness, forming a continuous membrane system. To view the spiral stair structure more clearly, 3D structure of the cisternae stack along the midline (yellow dashed lines and arrows in Figures 2B and 2C) of the cisternae was reconstructed (right in Figure 2E and Video S2), which further reinforced that the membrane twisted as helicoids resembling spiral stairs, similar to the structural motif of RER. The ratio of right- and left-handed RER-like cisternae stacks was counted to be approximately 1:1, independent of *Φ*_ethanol_ (Figure 2F). In RER, the right- and left-handed connections shared the same ratio (Marshall, 2013).

Video S1. 3D Reconstruction of a Right- and Left-Handed Single Helicoidal Cisternae Stack from the Confocal Fluorescence Images in z Stacks as Presented in Figures 2B and 2C, Related to Figure 2

Video S2. 3D Reconstruction of a Right- and Left-handed Single Helicoidal Cisternae Stack along the Midline of the Cisternae in Stacks as Indicated by the Yellow Dashed Arrow in Figures 2B and 2C, Related to Figure 2

In addition to the above demonstrated RER-like single helicoidal shape, cisternae stacks composed of multiple helicoids with two or more helicoids in one cisternae stack were also observed (Figures 3 and S8). Figures 3A–3C presented typical fluorescence images from the z stacks for the reconstruction of double, triple, and quad helicoidal membrane structures, respectively. Similar to the condition for single helicoid, the cisternae in the images with small panel numbers piled shoulder by shoulder (panel 1 in Figures 3A–3C), and then disconnected with the increase in panel numbers (panels 3 and 4 in Figures 3A–3C). The difference was, in the subsequent panel numbers, the disconnected cisternae (s) in one side failed to connect with the opposite part of the neighboring cisternae (s+1) as the case for single helicoid (Figure 2D), but connected to those cisternae with an interval of one cisterna (double helicoid in Figure 3A, where the left part of cisterna I connected with the right part of cisterna III as seen in panel 5), two cisternae (triple helicoid in Figure 3B, where the left part of cisterna I connected with the right part of cisterna IV as seen in panel 6), three cisternae (quad helicoid in Figure 3C, where the left part of cisterna I connected with the right part of cisterna V as seen in panel 6), four cisternae (penta helicoid in Figure S8), or five cisternae (hexa helicoid in Figure S8). In brief, for a multiple helicoidal structure with *n* helicoids, an interval of *n*-1 cisternae can be observed between two connected cisternae (Figure S9), the validity of which was further verified by the perfect reproduction of the observed experimental results in the serial sections of real helicoidal models (Figure S10). The morphology of the cisternae stacks with multiple helicoidal structural motifs was clearly illustrated from the 3D reconstructions in Figure 3D and Video S3. The helicoids in one cisternae stack were also connected with each other to form continuous membrane systems with interconnected single lumens, as indicated by the solid yellow arrows in Figures 3A–3C.

Video S3. 3D Reconstruction of Double, Triple, and Quad Helicoidal Cisternae Stack from the Confocal Fluorescence Images in z Stacks as Presented in Figures 3A–3C, Related to Figure 3

### Helicoids Formation Mechanisms

For samples incorporated with 30% DMPS, when water (25°C) was used for the reorganization of stacked bicelles (*Φ*_ethanol_ = 50% for the formation) to cisternae stacks, the average percentages of cisternae stacks with different *n* were 6% (*n* = 0, grana-like cisternae stacks), 34% (*n* = 1), 41% (*n* = 2), and 19% (*n* ≥ 3), respectively (Figure 3E). However, if the conditions for stacked bicelles formation or their reorganization to cisternae stacks were changed, the percentages can be varied (Figures 3E, 3F, and S11–S15). From the statistical analysis of the cisternae stacks samples formed at different conditions, we established the contribution of increased membrane electrostatics and decreased membrane bending elasticity to drive the formation of helicoidal cisternae stacks with increased *n*. With regard to membrane bending elasticity, higher temperature of the reorganization process that can soften lipid bilayers resulted in relatively larger *n* (Figure S11A), whereas the incorporation of phospholipids that can stiffen membranes (Figure S12), such as negatively charged 1,2-dipalmitoyl-*sn*-glycero-3-phospho-(1′-rac-glycerol) (sodium salt) or zwitterionic 1,2-distearoyl-*sn*-glycero-3-phosphocholine, caused smaller *n*. With regard to membrane electrostatics, parameters that cause the accumulation of electrostatic repulsive energy, such as increased percentages of negatively charged DMPS (Figures 3E and S13) or positively charged 1,2-di-(9Z-octadecenoyl)-3-trimethylammonium-propane (chloride salt) (Figure S14) resulted in larger *n*, whereas solvent conditions of NaCl solution, CaCl_2_ solution, or ethanol-water mixture that can screen electrostatic forces resulted in smaller *n* (Figure 3F). The electrostatics can also be readily altered by simply changing the solution pH. The average *n* initially decreased with the solution pH value from 7.0 to 3.6, and then increased from 3.6 to 2.0 (Figure S15). Minimum *n* was obtained at pH = 3.6, around the isoelectric point of phosphocholine (PC)-phosphatidylserine (PS) membrane system (Naumowicz and Figaszewski, 2014), which confirmed the important role of membrane electrostatics in forming helicoids. The ethanol volume percentage *Φ*_ethanol_ of the ethanol-water solution for bicelles formation also influenced the percentages of different morphologies in the cisternae stacks. With higher *Φ*_ethanol_, the average *n* increased (Figure S16) due to the following two reasons. First, with increased *Φ*_ethanol_, more ethanol molecules were incorporated in the lipid assemblies (Ly and Longo, 2004, Vanegas et al., 2010), which softened the lipid bilayer to facilitate their morphology transformation. Second, with increased *Φ*_ethanol_, thicker bicelles were formed, which caused more accumulated electrostatic repulsive force to drive the transformation of bicelles to cisternae stacks with increased *n* (Figure S17).

Taken together, membrane electrostatics drove the formation of helicoidal cisternae stacks with increased *n* against the resistance of membrane bending elasticity to deform the membranes (Figure 3E). The decreased electrostatic repulsive energy *W*_e_ of helicoidal structures might be responsible for the formation of helicoids with different *n*. For two parallel surfaces in the helicoid with angle *θ* between the axis and local ramp plane (Figure 3F), *W*_e_ was given as $W_{\text{e}}=-\int_{\infty }^{Z}P\left(Z\right)d_{Z}$, where *z* was the distance between the two surfaces and *P*(*z*) was the lateral pressure expressed as *P*(*Z*) = 2*ɛ*_0_*ɛ*_*s*_*K*^2^(*kT*/*e*)^2^*sin*(*θ*), with *ɛ*_0_, *ɛ*_s_, *k*, *T e*, and *K,* respectively, representing dielectric constant of vacuum, dielectric constant of solvent, Boltzmann constant, temperature, elementary electron charge, and a parameter related to charge density (Israelachvili, 2015). With the decrease of *θ*, *P* (*z*) decreased, so smaller *W*_e_ was obtained. Therefore, flat surface (*θ* = 90°) tended to transform to helicoid (*θ* < 90°) to decrease *W*_e_. The formation of multiple helicoids might be related to the transient volume-confined state at the sudden variation of solvent condition. Obviously, with fixed number of lipid bilayers in fixed confined space, multiple helicoids tended to form to achieve smaller *θ*, namely, more tilted ramps, for lowest *W*_e_. To further decrease the electrostatic energy, secondary helical structures tended to form (Figure 3G), mainly from long and fully expanded helicoidal cisternae stacks, which were easier to twist compared with cisternae stacks with smaller expansion extent. The reorganization of stacked bicelles to helicoidal cisternae stacks might occur in three stages (Figure S18): the fusion of lipid bilayers to form cisternae driven by edge energy (stage 1), the breaking of cisternae to fragmented small helicoids (stage 2), and the fusion of fragmented helicoids to form continuous helicoidal cisternae stacks (stage 3). These three stages might also happen simultaneously.

Charged lipids were the constitutive components of cellular membranes. In RER, the anionic phospholipids such as PS and phosphatidylinositol constituted over 10% of the total lipids (Mato, 1990, van Meer et al., 2008). Moreover, the RER sheets were often spatially confined by the cytoplasmic matrix. According to our experimental and calculation results, RER in cells might be helicoidally arranged to decrease interbilayer electrostatic repulsive energy in confined space. Moreover, although not yet reported in RER, multiple helicoids might exist in cells (Figure S19).

### Modulated Enzyme Crowding and De-crowding in “Breathing” Cisternae Stacks

**Figure 4:**
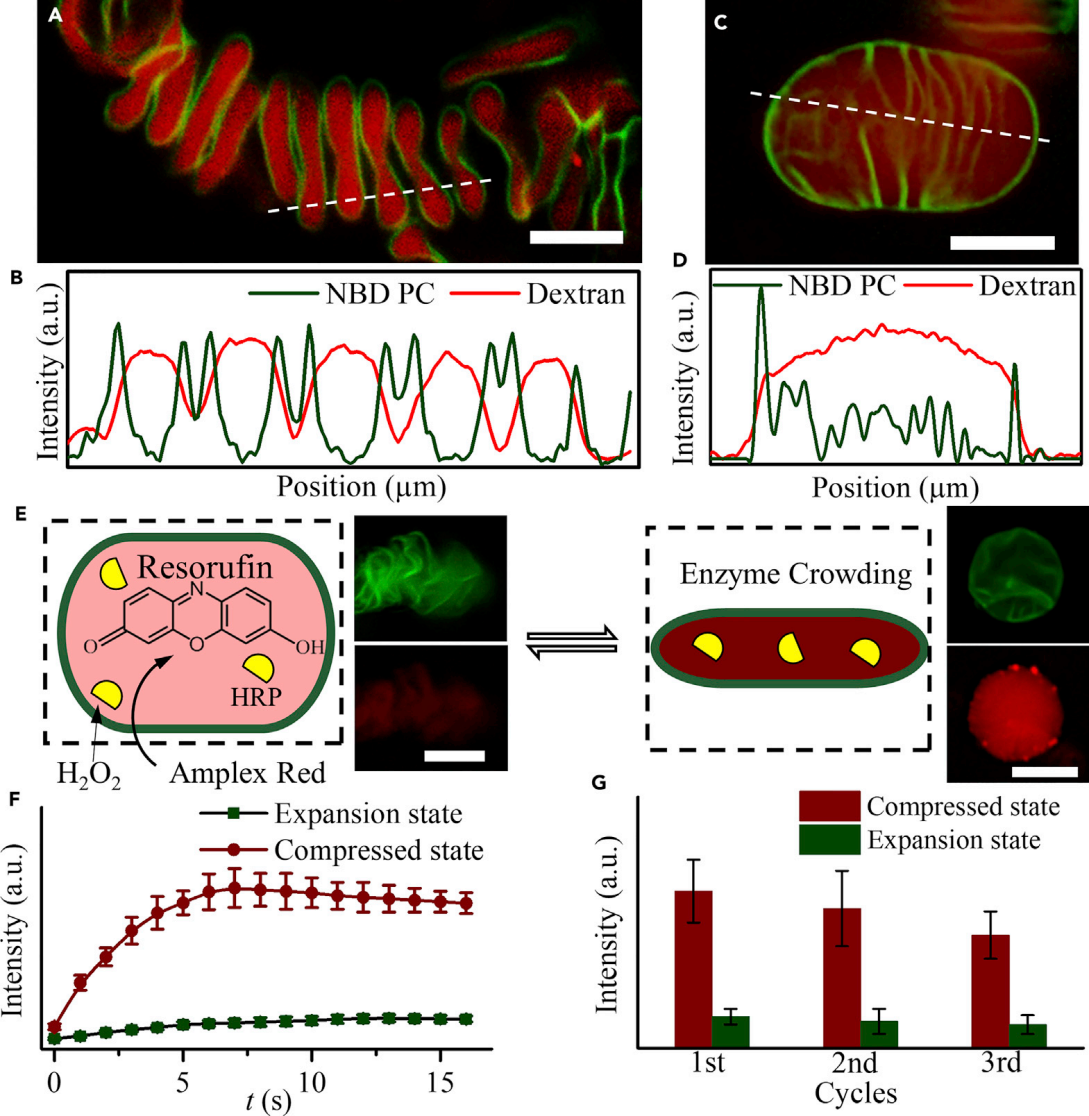
Breathing” Modulated Enzyme Catalytic Process in the Helicoidal Cisternae Stacks (A and C) Fluorescence image of the helicoidal cisternae stack (A) and ellipsoidal liposome (C) encapsulated with Rhodamine B isothiocyanate-Dextran (∼70 kDa). (B and D) The profile of the fluorescence intensity along the (B) white dashed line in (A) and (D) white dashed line in (C). (E) Schematic illustration of the enzyme catalytic reactions in the helicoidal cisternae stacks and typical fluorescence images of the cisternae stacks in different states encapsulated with fluorescent products. (F) Variation of the fluorescence intensity of the fluorescent product resorufin with time for cisternae stacks in expansion state (green curve) and compressed state (red curve), respectively. (G) Variation of the maximum fluorescence intensity of the reaction product resorufin with cycle numbers in compressed (red) and expanded (green) cisternae stacks. Scale bars, 10 μm. Error bars are ±SEM. See also Figures S20–S22.

The entrapment of biomolecules during the reorganization process demonstrated quite low loading efficiency of molecules (Figure S4) because of the quick reorganization process from bicelles to cisternae stacks. To functionalize the helicoidal cisternae stacks for potential proto-organelle models, a method for high loading efficiency of molecules was desired. Here, we proposed a way for loading of molecules through the transient membrane defects generated in an exocytosis-like process, mimicking the active secretion process in RER, caused by the binding of free avidin to the cisternae stacks incorporated with 1,2-dipalmitoyl-sn-glycero-3-phosphoethanolamine -N-(cap biotinyl) (sodium salt) (16:0 Biotinyl Cap PE) (Figure S20). The concentration of avidin influenced the loading efficiency and the morphology of the cisternae stacks. When *C*_avidin_ was below 0.02 mg/mL, the increase of *C*_avidin_ resulted in the encapsulation of more fluorescent molecules, without influencing the helicoidal structure of the cisternae stacks (Figure S21). However, when *C*_avidin_ was above 0.05 mg/mL, the cisternae in the stacks fused shoulder by shoulder, forming ellipsoidal liposomes with interconnected inner membrane system, similar to the morphology of some kinds of mitochondria. According to the fluorescence images and fluorescence intensity profiles, the fluorescent molecules were uniformly distributed in the cisternae stacks or ellipsoidal liposomes (Figures 4A–4D). No obvious leakage of fluorescent molecules was observed after several days. The detached “exosomes” encapsulated with high concentration of fluorescent molecules during the exocytosis-like process were observed as red spots sitting on the membrane surface or aggregates in solution (Figures S20B and S20C).

The stacked cisternal structures in cells often displayed “breathing”-like expansion and compression for specific functions. The RER-like helicoidal structures can also “breathe” under the stimuli of external solvent condition (Figure S22). In 150 mM PBS, the cisternae stacks compressed to densely packed membranes with rough surface because of the screening of interbilayer electrostatic repulsive forces, and consequently, fluorescent molecules in them were concentrated. When re-dispersed in water, they restored to loosely packed state, with re-diluted fluorescent molecules. The interbilayer forces (van der Waals force, hydration force, and electrostatic repulsive force) among lipid bilayers and osmotic force were the main driving force for the “breathing.” Motivated by the “breathing” property of the cisternae stacks to concentrate or dilute molecules, an adaptive proto-organelle model from external solvent condition was proposed by trapping horseradish peroxidase in the cisternae stacks (Figure 4E). Several seconds after the addition of H_2_O_2_ and Amplex Red, the typical red fluorescence of resorufin (reaction product) was monitored under the fluorescence microscope (Figure 4E), both for expanded cisternae stacks in water and compressed cisternae stacks in PBS. Because of the relatively concentrated enzyme environment compared with expanded cisternae stacks, quicker increase of fluorescence intensity to higher fluorescence level was observed in cisternae stacks in compressed state (Figure 4F). At least three cycles of luminal-space-modulated enzyme catalytic processes were realized in the “breathing” cisternae stacks, with slight decrease in the measured fluorescence intensities with increased cycle numbers (Figure 4G). In cells, for higher efficiency and better timeliness, most enzyme-catalyzed reactions occurred in molecularly crowded or confined environments. The helicoidal cisternae stacks with “breathing” lumens here can provide robust model to study these enzyme catalytic reactions, especially in some sophisticated systems involving cascade reactions.

In summary, we formed RER-like helicoidal cisternae stacks from charged stacked bicelles and proposed an adaptive proto-organelle model for molecules crowding. Membrane electrostatics controlled the formation of helicoidal cisternae stacks with different *n* to decrease the electrostatic repulsive energy among lipid bilayers, which might be the possible shaping mechanism for the helicoidal RER in cells. The RER-like cisternae stacks can reversibly expand and compress, which endowed them the ability to be applied in many volume-confined processes. Moreover, except for RER-like helicoidal cisternae stacks, many other novel and advanced membrane models were also provided. This work provided possible organelle shaping mechanisms and advanced membrane models for biomimetics or cell biology.

### Limitation of Study

The helicoidal cisternae stacks samples were mixtures of lipid assemblies with different morphologies. Although the percentages can be easily modulated, it is still difficult to form samples with one morphology.

## Methods

All methods can be found in the accompanying Transparent Methods supplemental file.

## Supplemental Information

Document S1. Transparent Methods and Figures S1–S22
