# Supplementary material for: Self-Assembled Rough Endoplasmic Reticulum-Like Proto-Organelles
Source: iScience. 2018 Sep 27;8:138–47. doi: 10.1016/j.isci.2018.09.020 (PMC6180236; doi:10.1016/j.isci.2018.09.020)
Supplement: Document S1. Transparent Methods and Figures S1–S22 [file mmc1.pdf]

ISCI, Volume *8*

Supplemental Information

Self-Assembled Rough Endoplasmic Reticulum-Like Proto-Organelles

Qingchuan Li and Xiaojun Han

Supplemental Data Items

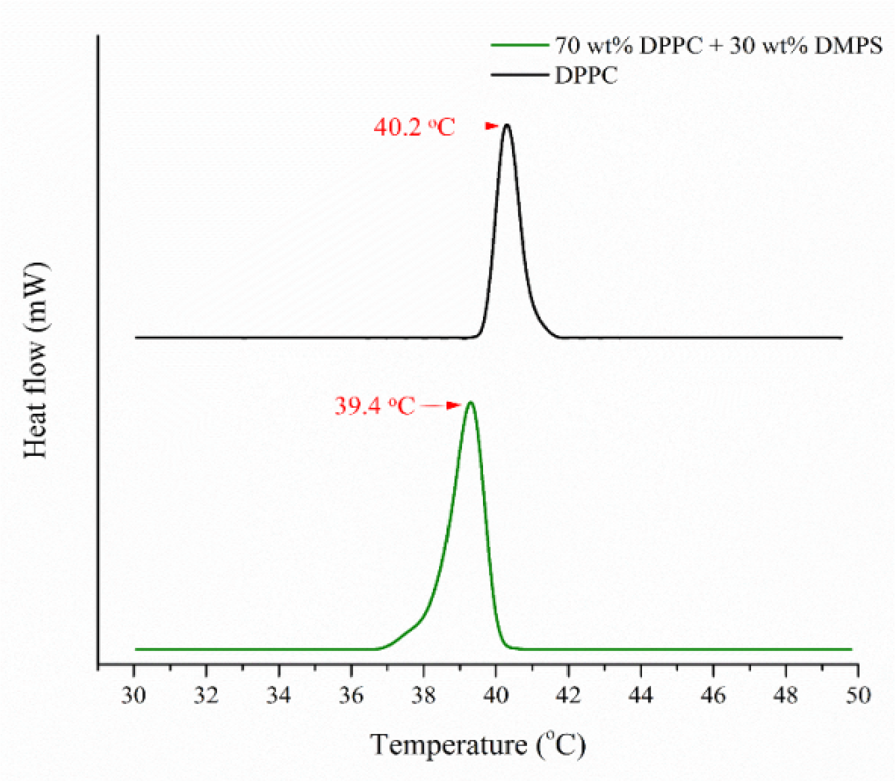

Figure S1. Endothermic calorimetric thermogram, related to Figure 1

Endothermic calorimetric thermogram of stacked bicelles formed from DPPC and DPPC/DMPS (7/3, *w*/*w*) mixtures. The stacked bicelles were formed in solution with lipid concentration of 0.10 mg/mL and ethanol volume percentage of 50%.

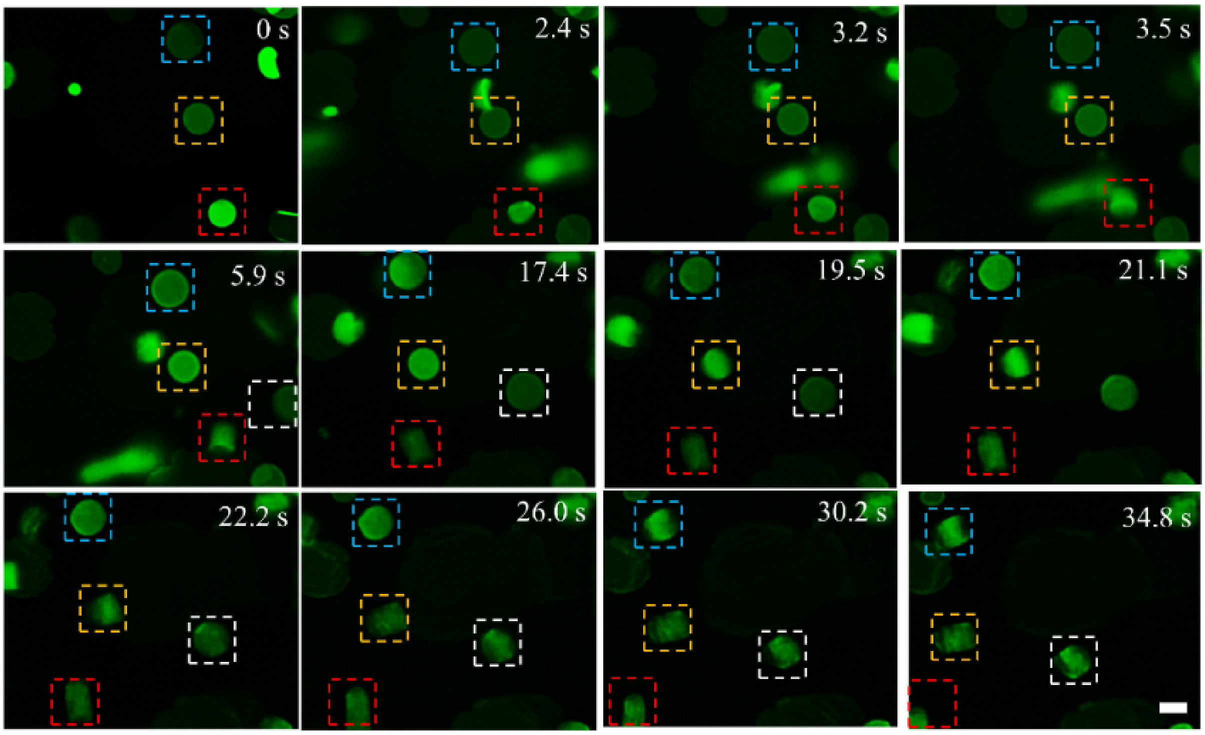

Figure S2. Fluorescence images with time, related to Figure 1

Fluorescence images for the transformation process from stacked bicelles to cisternae stacks. The dash boxes with different colors represent different samples. The scale bar was 10 μm.

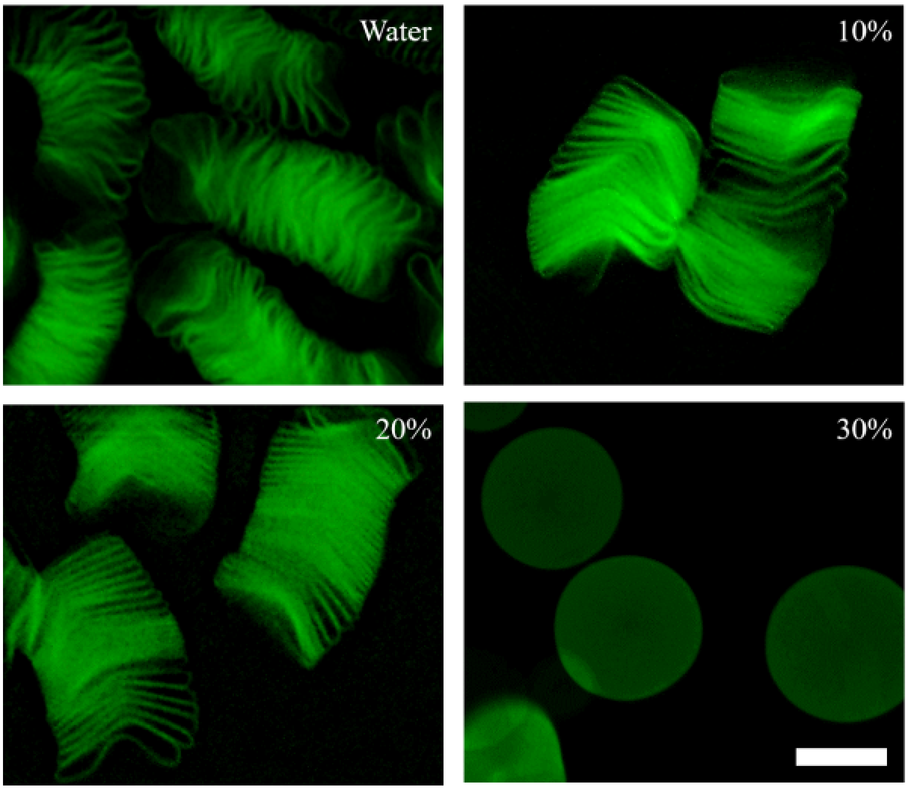

Figure S3. Fluorescence images, related to Figure 1

Fluorescence images of the samples after the stacked bicelles were dispersed into ethanol-water solution with different ethanol volume percentage. The stacked bicelles were formed in 50% ethanol solution from DPPC/DMPS (7/3, w/w). The scale bar was 10 μm.

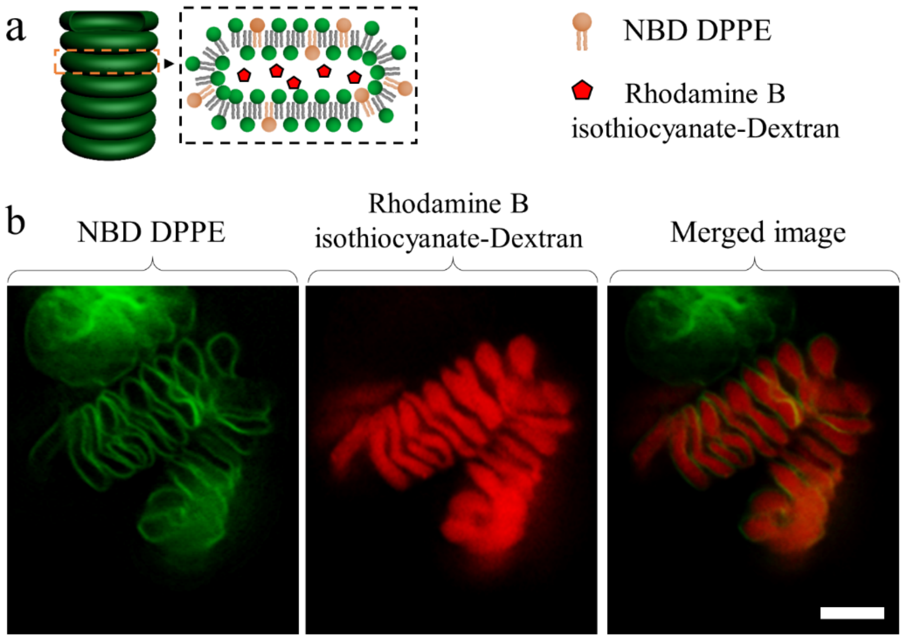

Figure S4. The entrapment of water-soluble fluorescent markers in cisternae stacks during the reorganization process, related to Figure 1

a, Schematic illustration of the entrapment experiments. b, Fluorescence images of the cisternae stacks indicating the encapsulation of fluorescent molecules (red) in the stacks (green). The scale bar was 10 μm.

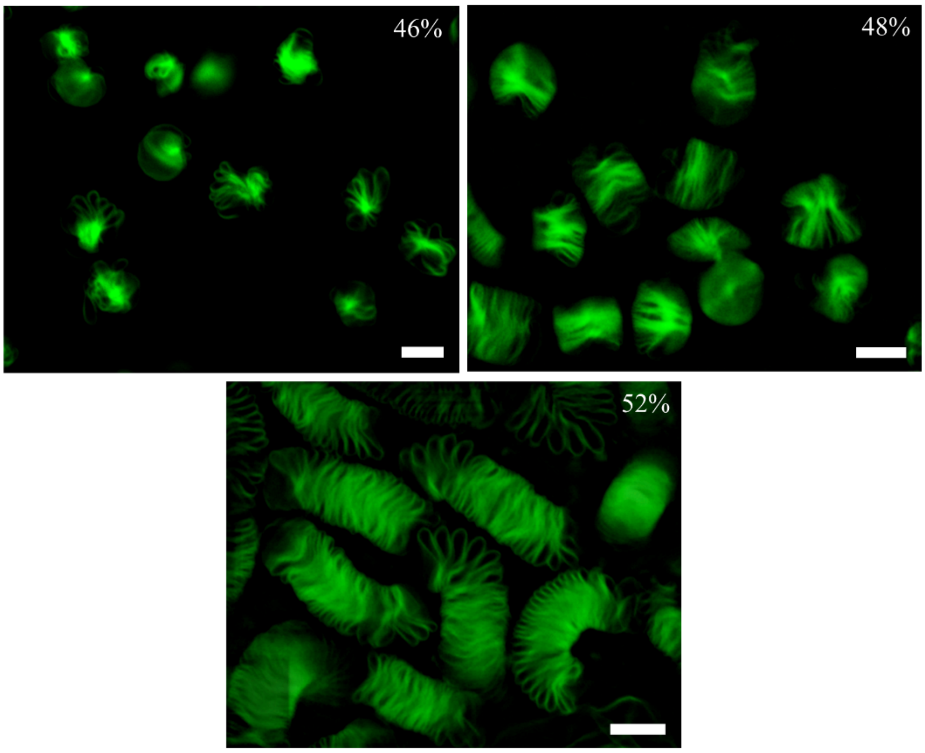

Figure S5. Fluorescence images, related to Figure 1

Fluorescence images of cisternae stacks in water transformed from stacked bicelles formed in different $\Phi_{ethanol}$. The scale bars were 10 μm.

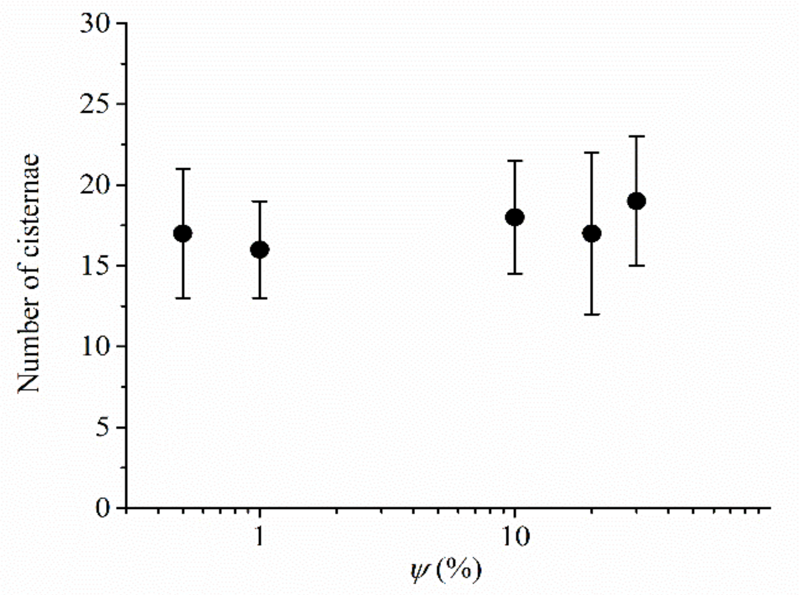

Figure S6. The number of cisternae in single stacks, related to Figure 1

Influence of the percentage of negatively charged DMPS ψ in stacked bicelles on the number of cisternae in the cisternae stacks. The stacked bicelles were formed in solution with lipid concentration of 0.10 mg/mL and ethanol volume percentage of 50%. Error bars are ±SEM.

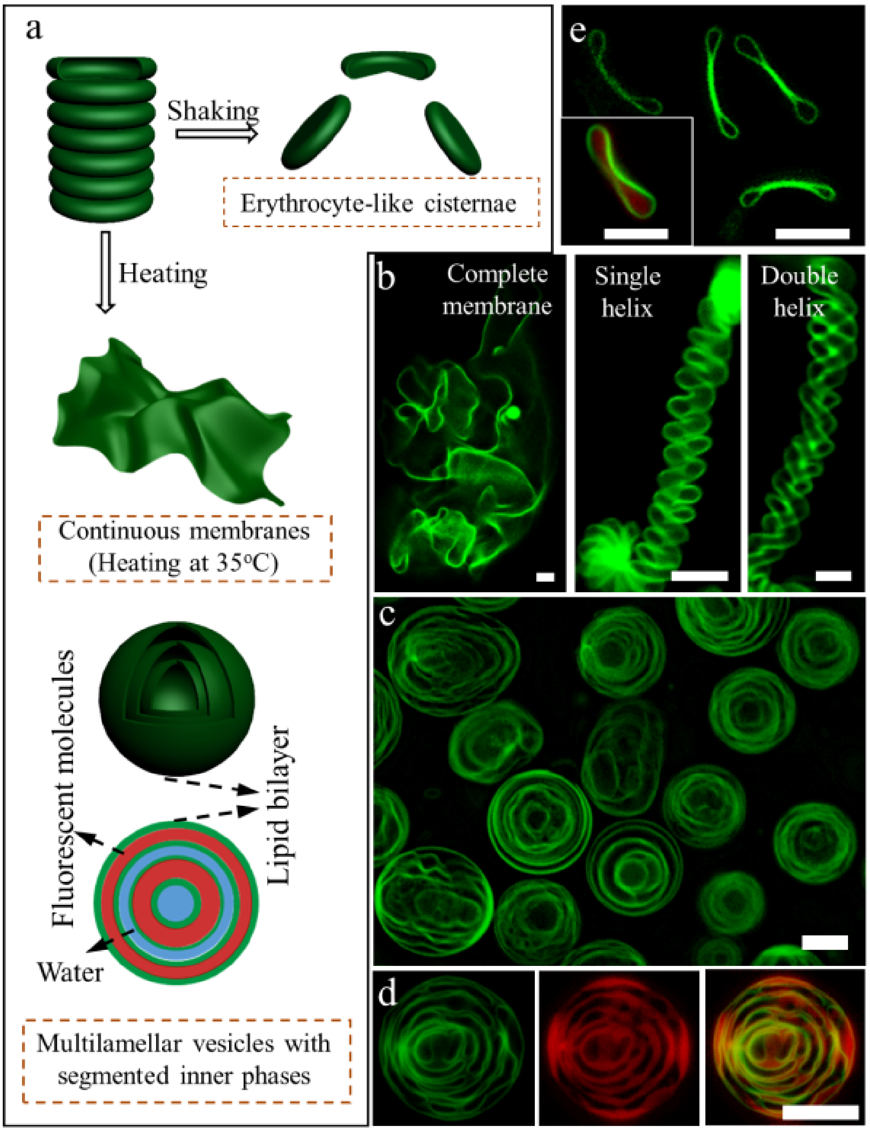

Figure S7. Morphology evolution of cisternae stacks, related to Figure 2
a, Schematic presentation of the morphology evolution of cisternae stacks treated with shaking or heating. b, Fluorescence image of the continuous membrane structures from cisternae stacks by heating the samples at 35°C for 12h. c, Fluorescence image of multilamellar vesicles formed by heating the cisternae stacks samples at 45°C. d, Fluorescence images of the multilamellar vesicles with spatially segmented inner phases. e, Fluorescence image of the erythrocyte-like separate cisternae formed by the vigorously shaking of the cisternae stacks samples. The inset in e is the fluorescence image of one cisterna encapsulated with Rhodamine B isothiocyanate-Dextran (~70 kDa). The scale bars were 10 μm. When heating at 45°C (above T_m), the cisternae stacks quickly transformed to multilamellar vesicles (Fig. S7c). Rhodamine B isothiocyanate-Dextrans (~70 kDa) added during the transformation can be encapsulated to form multilamellar vesicles with spatially segmented inner phases, namely, the contents followed the repeated pattern with water/Dextran in the segments (Fig. S7d). Vigorously shaking would break the cisternae stacks into separate cisternae with similar morphology to erythrocytes (Fig. S7e). During this process, Rhodamine B isothiocyanate-Dextrans can enter the cisternae and be encapsulated (The inset in Fig. S7e).

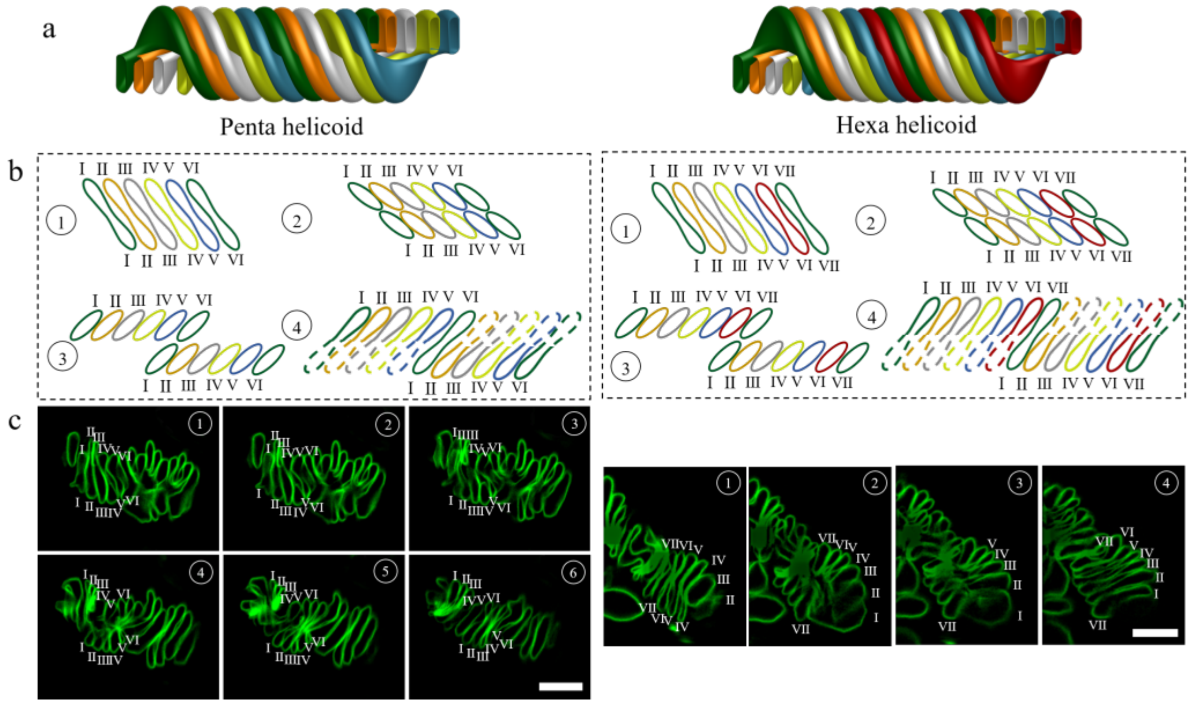

Figure S8. Formation of multiple helicoidal cisternae stacks with *n*=5 and *n*=6, related to Figure 3

a, Schematic representation of penta and hexa helicoidal cisternae stacks. b, Schematic illustration of the variation tendency of the cisternae stacks in Z-stacks under laser confocal fluorescence microscope for cisternae stacks with *n*=5 (left) and *n*=6 (right). c, Typical fluorescence images of the cisternae stacks with multiple helicoidal morphologies visualized in Z-stacks by laser confocal microscope: *n*=5 (left) and *n*=6 (right). The scale bars were 10 μm.

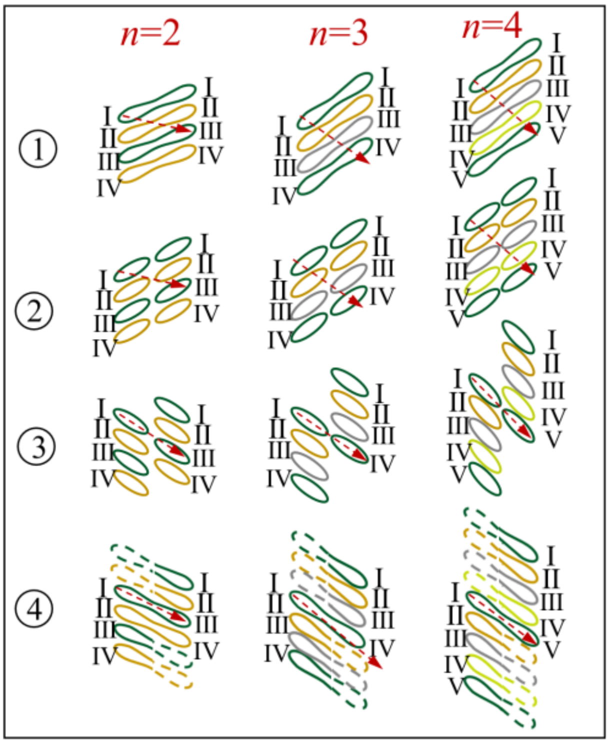

Figure S9. Schematic illustration, related to Figure 3

Schematic illustration of variation of the cisternae in Z-stacks under laser confocal fluorescence microscope for cisternae stacks with different number of helicoid *n*. Different color represented the cisternae from different helicoid in the multiple helicoidal cisternae stack. The dashed red arrows indicated the connection between cisternae.

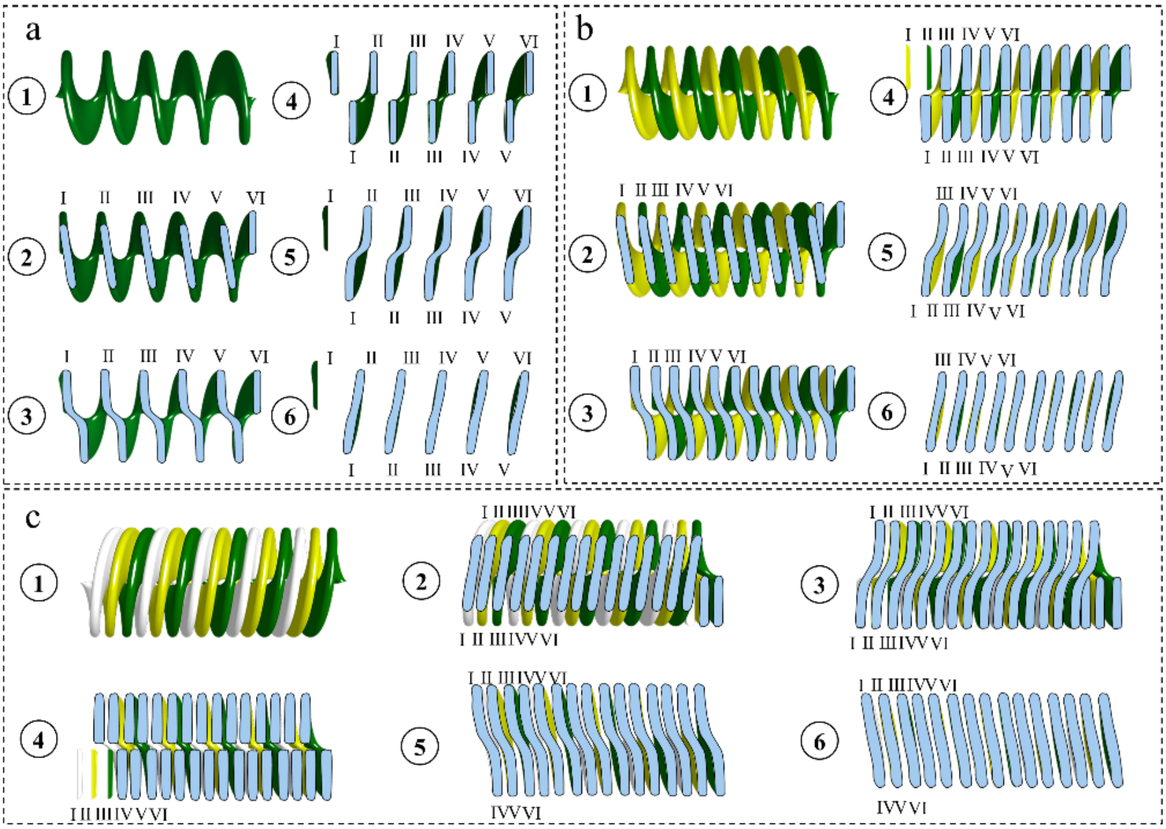

Figure S10. Serial sections, related to Figure 3

Serial sections of helicoidal models with different number of helicoid: (a) n=1, (b) n=2, (c) n=3.

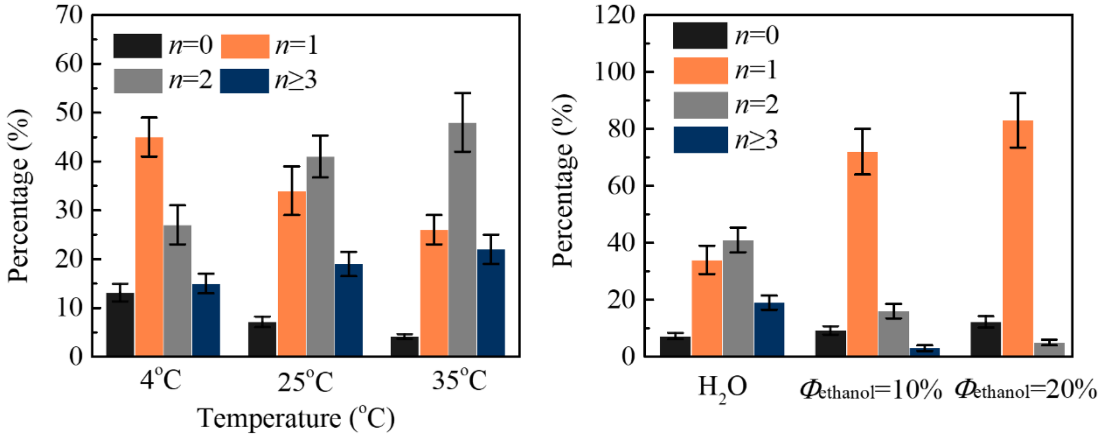

Figure S11. Statistical analysis, related to Figure 3

Influence of temperature (a) and solvent (b) conditions for the reorganization of DPPC/DMPS (7/3, *w*/*w*) bicelles to cisternae stacks on the percentages of helicoidal structures with different *n* in the samples. The stacked bicelles were formed in 50% ethanol solution. Error bars are ±SEM.

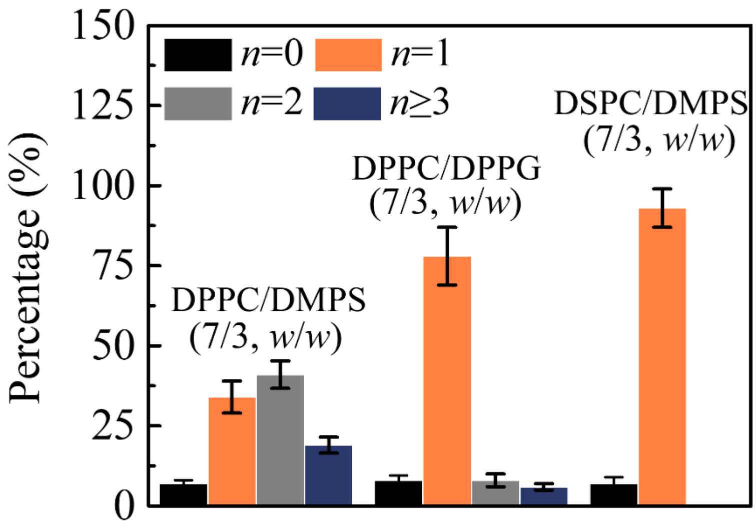

Figure S12. Statistical analysis, related to Figure 3
Influence of lipid compositions on the formation of helicoidal cisternae stacks with different *n* (*N*=100). Error bars are ±SEM.

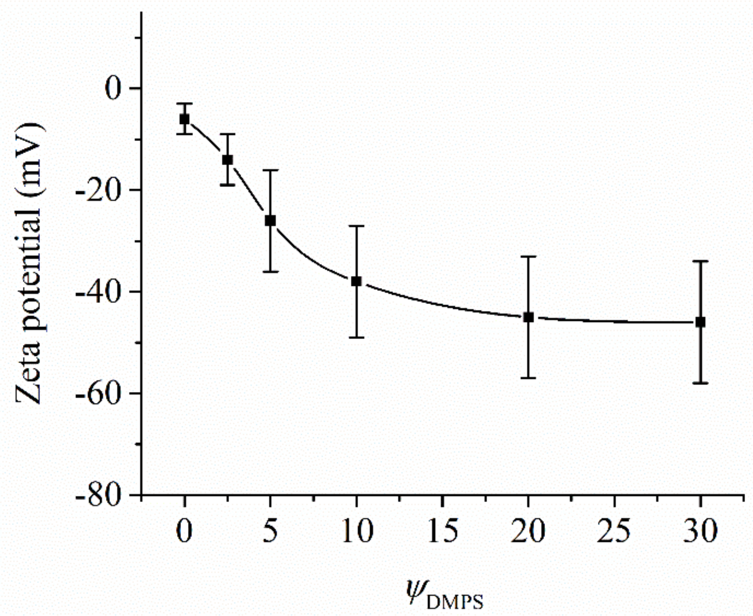

Figure S13. Influence of DMPS on the Zeta potential of helicoids, related to Figure 3
Variation of zeta potential of cisternae stacks in water with ψ_{DMPS}. Error bars are ±SEM.

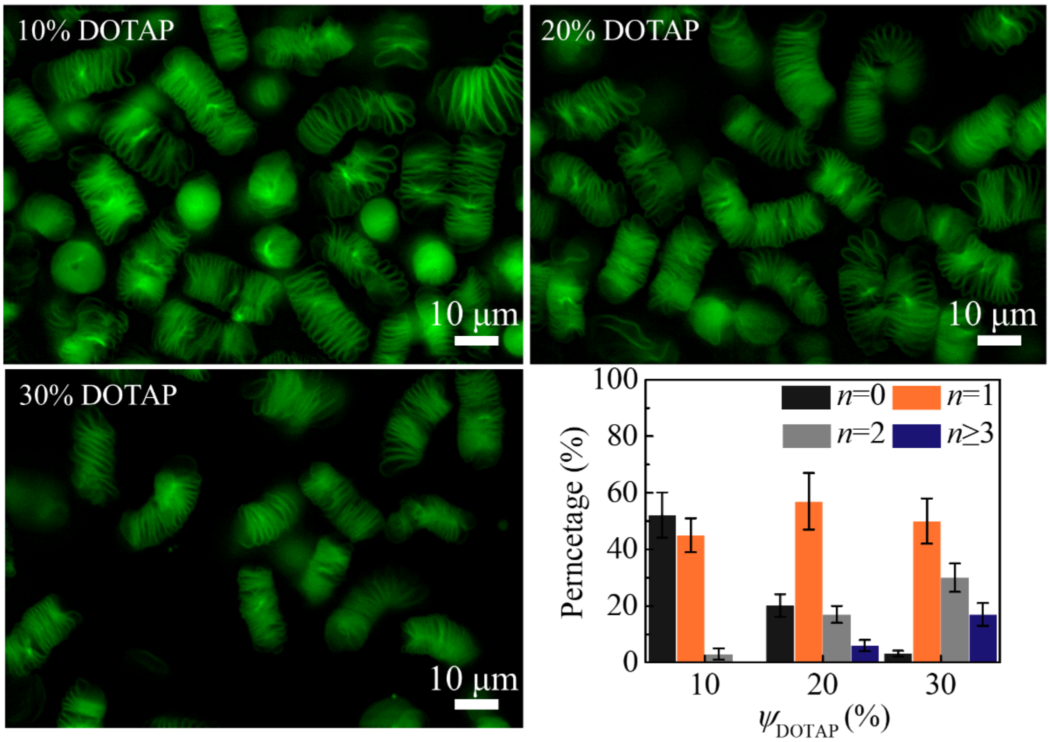

Figure S14. Fluorescence images and statistical analysis, related to Figure 3
Fluorescence images of DPPC/DOTAP cisternae stacks incorporated different percentages of DOTAP, and counted percentages of cisternae stacks with different *n* in the samples. Error bars are ±SEM. The scale bars were 10 μm.

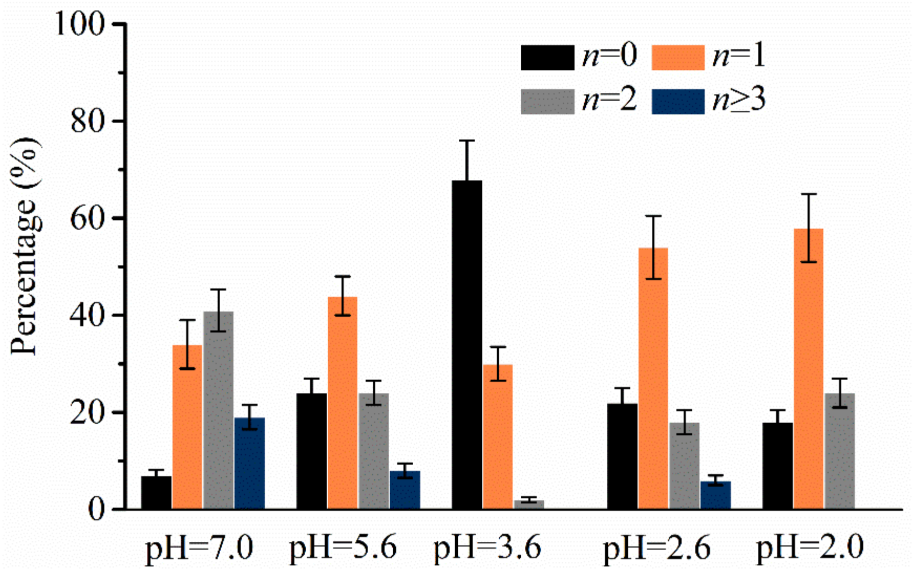

Figure S15. Influence of pH on the morphologies of lipid assemblies, related to Figure 3
Variation of the percentages of different morphologies in cisternae stacks with solution pH value. Error bars are ±SEM.

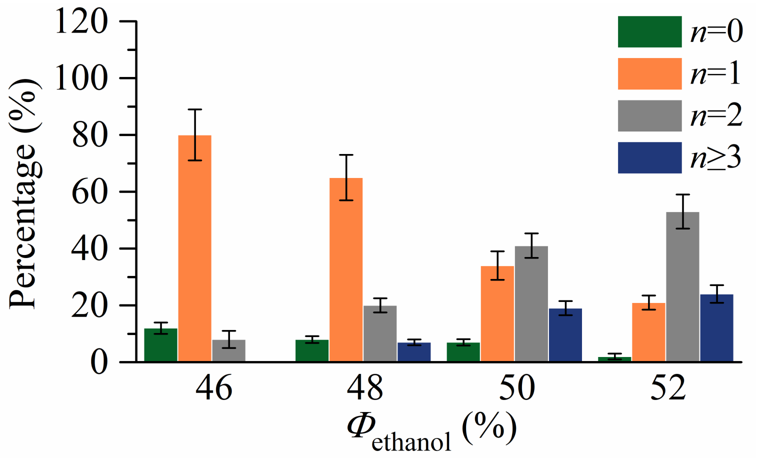

Figure S16. Statistical analysis, related to Figure 3

Influence of ethanol volume percentage $\Phi_{ethanol}$ for the formation of DPPC/DMPS (7/3, w/w) stacked bicelles on the percentages of cisternae stacks with diffetent *n* (*N*=200). Error bars are ±SEM.

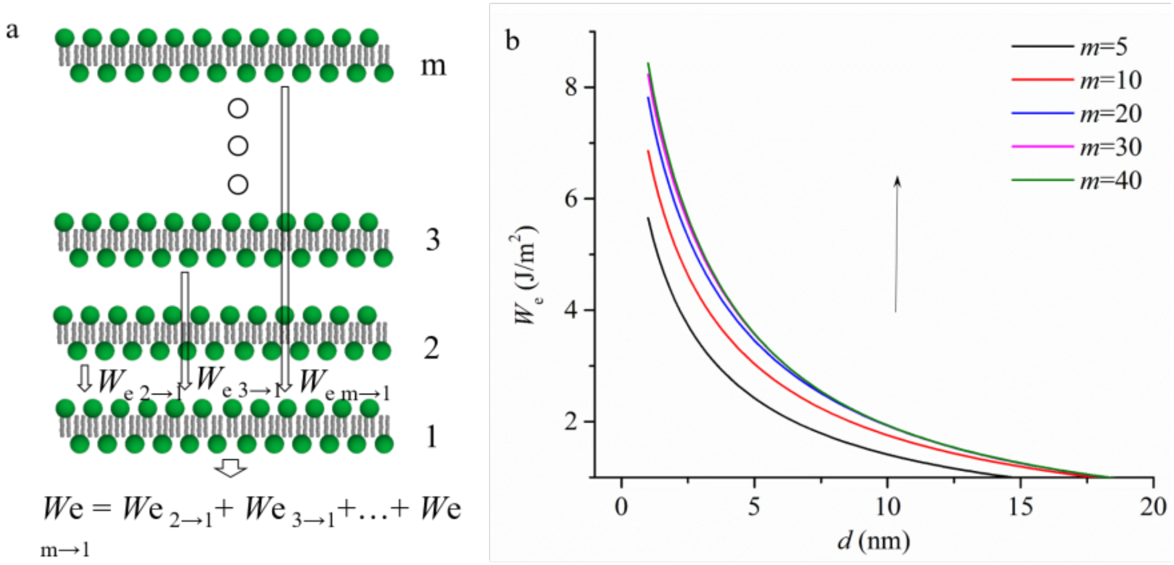

Figure S17. Interlayer energy calculation, related to Figure 3

The accumulation of electrostatic repulsive energy in the stacked bicelles with *m* lipid bilayers when they were dispersed in water. a, Schematic illustration of the accumulation of electrostatic repulsive energy on one lipid bilayer from other lipid bilayers in stacked bicelles. b, Calculated electrostatic repulsive energy W_e with *m*.

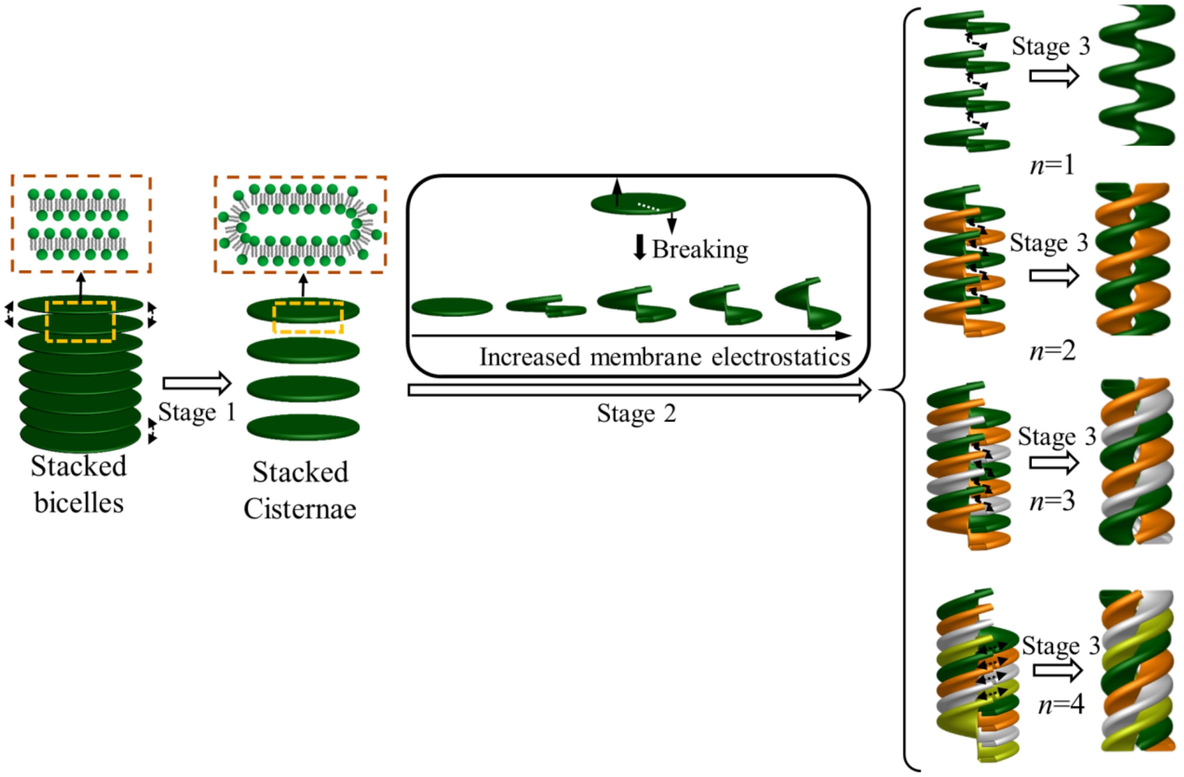

Figure S18. Schematic illustration, related to Figure 3

Schematic illustration for the reorganization of stacked bicelles to helicoidal cisternae stacks with different *n*.

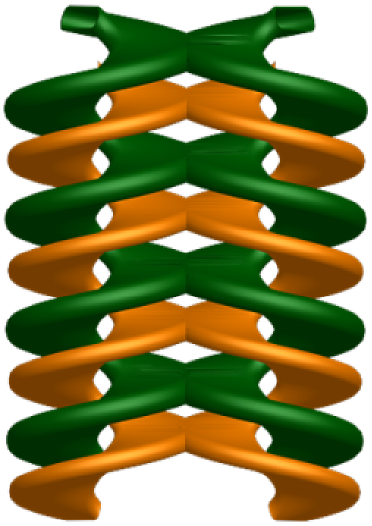

Figure S19. Anticipated membrane structures in RER, related to Figure 3
Schematic representation of double helicoidal model that might exist in natural RER.

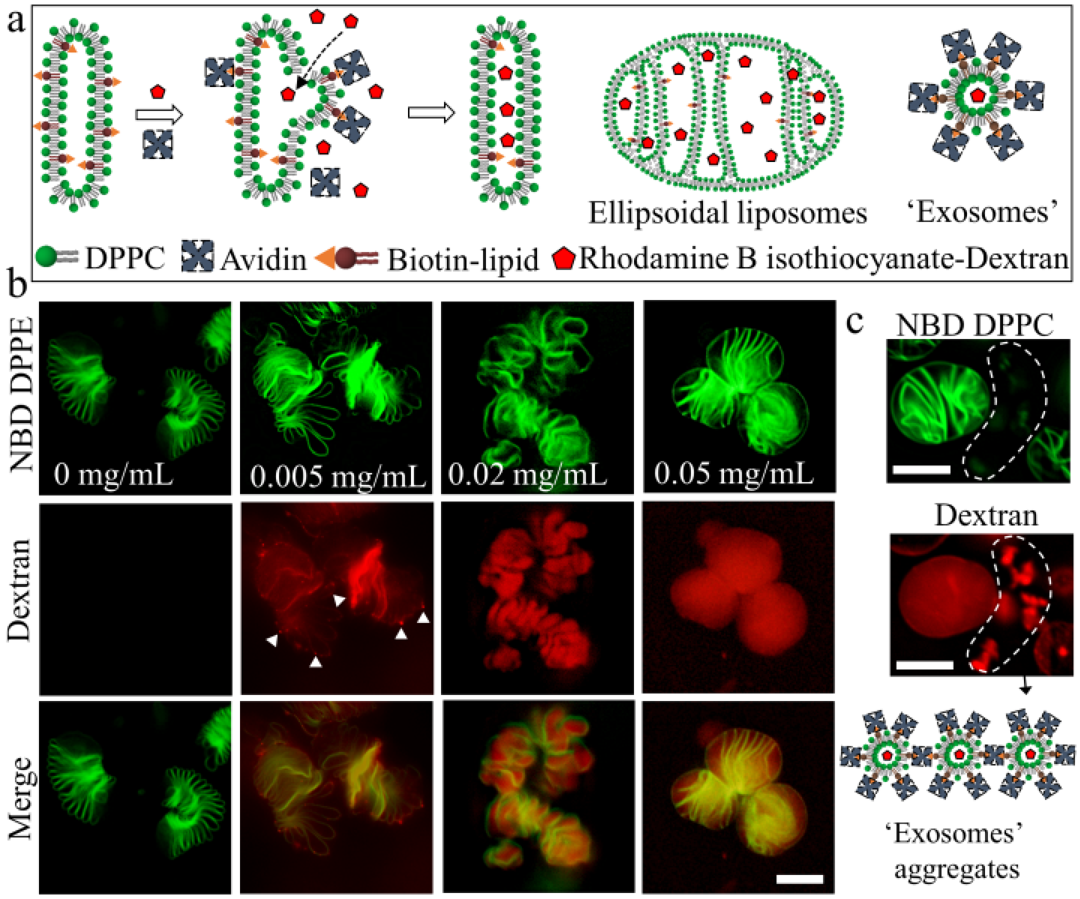

Figure S20. Loading of fluorescent molecules in the RER-like cisternae stacks, related to Figure 4

a, Schematic illustration of the entrapment of fluorescent molecules through the exocytosis-like process caused by the binding of avidin to cisternae stacks incorporated with Biotinyl Cap PE. b, Fluorescence images of samples composed of DPPC/DMPS/ Biotinyl Cap PE (*w*/*w*/*w*, 7/2/1) after incubating with avidin of different concentrations. The white arrow heads indicated the formation of liposomes on the membrane surface. c, Fluorescence images and schematic illustration for the formation of 'exosome' aggregates in solutions. The concentration of Rhodamine B isothiocyanate-Dextran (~70 kDa) was 0.01 mg/mL. The incubation time was fixed at 2h. The scale bars were 10 μm.

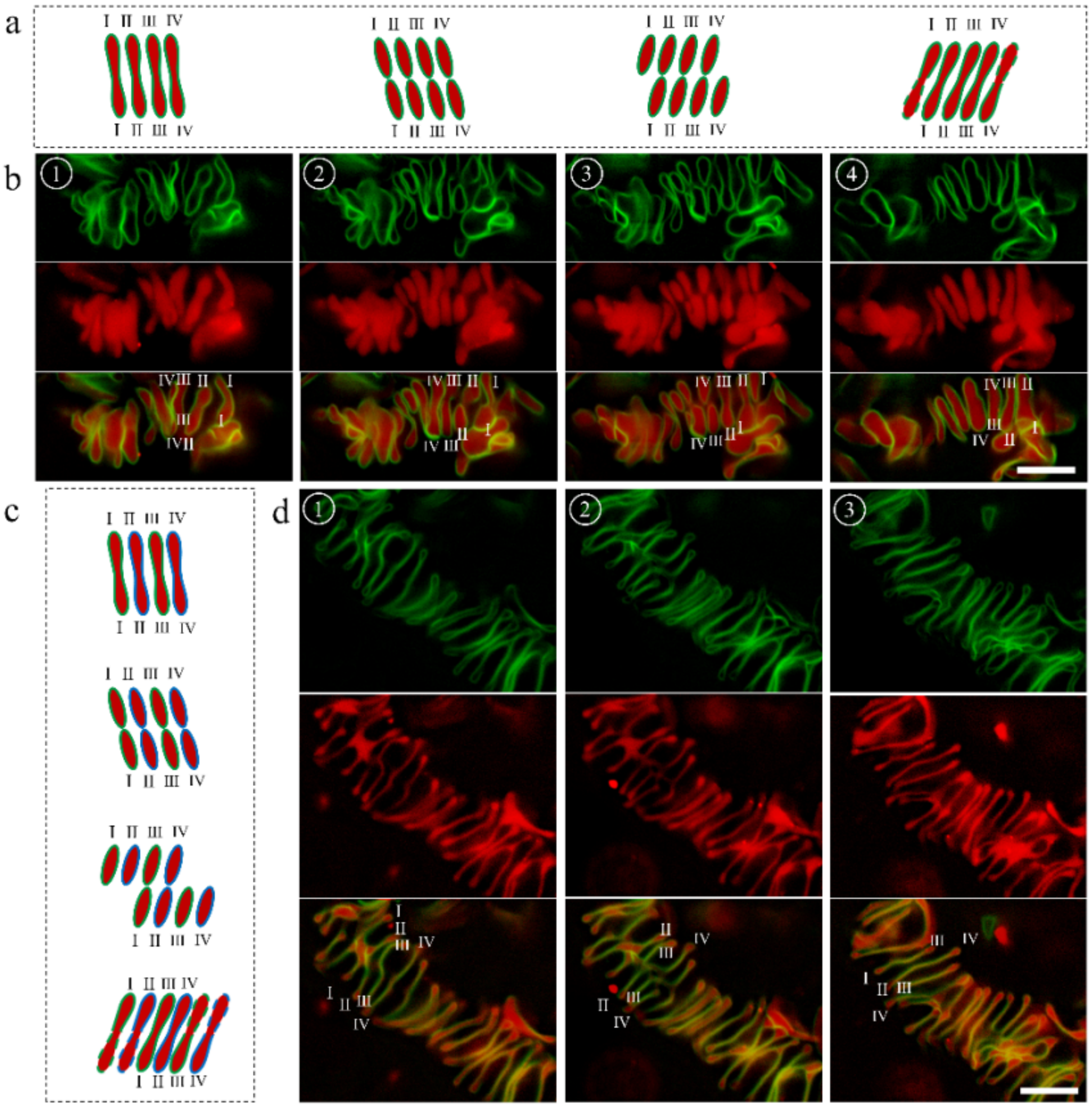

Figure S21. **Confirmation of the helicoidal structures after encapsulating Rhodamine B isothiocyanate-Dextran (~70 kDa), related to Figure 4**

a, Schematic illustration of the variation tendency of the RER-like helicoidal cisternae stacks in Z-stacks. b, Typical fluorescence images the RER-like cisternae stacks (green) encapsulated with fluorescent molecules (red) visualized in Z-stacks. c, Schematic illustration of the variation tendency of the double helicoidal cisternae stacks in Z-stacks. d, Typical fluorescence images the double helicoidal cisternae stacks (green) encapsulated with fluorescent molecules (red) visualized in Z-stacks. The Morphology of the cisternae stacks were determined according to Fig. S9 as labelled in Fig. S21b, d. The scale bars were 10 μm.

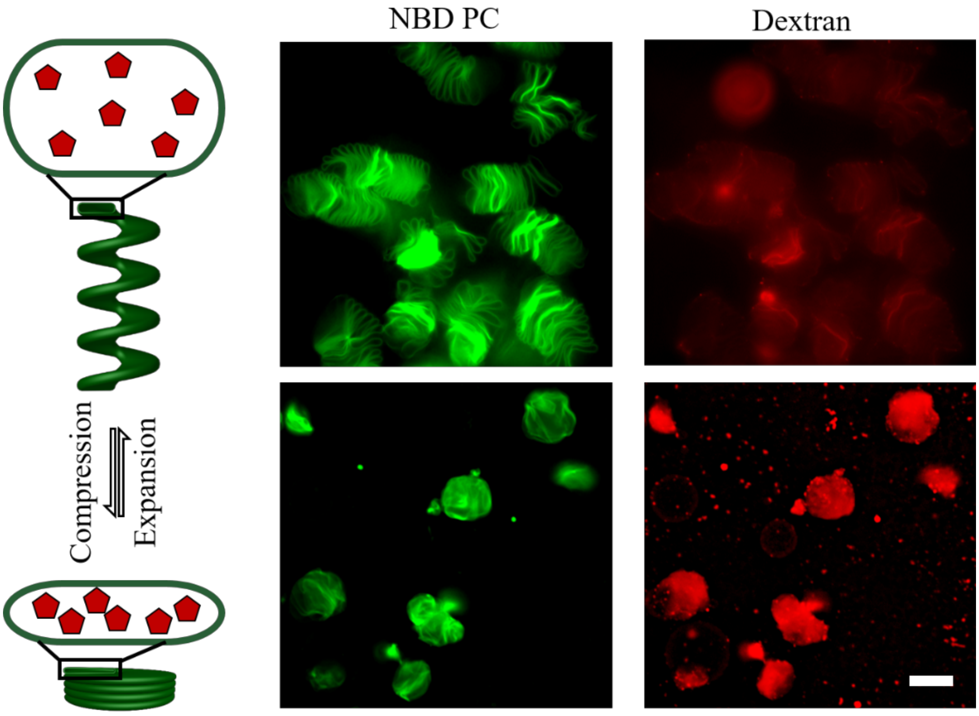

Figure S22. “Breathing” cisternae stacks, related to Figure 4

Schematic illustration of “breathing” process of helicoidal cisternae stacks to realize the reversible concentration and dilution of dextrans, and typical fluorescence images the expanded cisternae stacks (top) in water and compressed cisternae stacks (bottom) in 150 mM PBS encapsulated with Rhodamine B isothiocyanate-Dextran (~70 kDa) (red). The scale bar was 10 μm.

Transparent Methods

Experimental Procedures

Materials 1,2-dipalmitoyl-*sn*-glycero-3-phosphocholine (DPPC), 1,2-distearoyl-*sn*-glycero-3 -phosphocholine (DSPC), 1,2-dimyristoyl-*sn*-glycero-3-phospho-L-serine (sodium salt) (DMPS), 1,2-dipalmitoyl-*sn*-glycero-3-phospho-(1'-*rac*-glycerol) (sodium salt) (DPPG), 1,2-dioleoyl -3-trimethylammonium-propane (chloride salt) (DOTAP), 1,2-dipalmitoyl-sn-glycero-3 -phosphoethanolamine-N-(cap biotinyl) (sodium salt) (16:0 Biotinyl Cap PE), 1-Myristoyl-2-[12-[(7-nitro-2-1,3-benzoxadiazol-4-yl)amino]dodecanoyl]-*sn*-Glycero-3-Phosphocholine (NBD PC) were purchased from Avanti Polar Lipids (USA). Texas red labelled 1,2-dihexadecanoyl-*sn*-glycero-3-phosphoethanolamine, triethylammonium salt (TR-DHPE) was obtained from Invitrogen (China). Avidin from egg white, peroxidase from horseradish (HRP), Amplex Red, hydrogen peroxide, fluorescein isothiocyanate (FITC)-labelled dextrans (150 kDa), Rhodamine B isothiocyanate-Dextran (70 kDa), ethanol, sodium chloride, magnesium chloride, disodium hydrogen phosphate, and sodium dihydrogen phosphate were purchased from Sigma (China). Millipore Milli-Q water with a resistivity of 18.2 MΩ cm was used in the experiment.

Formation of microsized stacked bicelles incorporated with charged lipids. Stacked bicelles were prepared according to our previously described procedure with little modification(27). Briefly, 10 μL of lipid solution (5.0 mg/mL in ethanol) was placed in a centrifuge tube and dried under N_2 stream. Then the lipids were re-dissolved in ethanol (V_1) and water (V_2) mixture solution (V_1+V_2=500 μL). Afterwards, the mixture was sealed in a cell with thickness of 1.5 cm. The temperature of the cell was controlled with a Linkam PE120 heat stage: 50ºC for 30min, and slow decrease to 25ºC at a rate of 0.5ºC/min. To form stacked bicelles in different ethanol volume percentage Φ_{ethanol}, the ratio of V_1 to V_2 was modulated. To form stacked bicelles incorporated with charged lipids, the composition of the lipid solution was varied with fixed lipid concentration of 0.10 mg/mL. 1.0% NBD PC or 0.5% TR-DHPE was labelled in the cisternae stacks for the observation under microscope.

Formation of helicoidal cisternae stacks from stacked bicelles. Helicoidal cisternae stacks were formed from stacked bicelles by replacing the solvent with pure water or ethanol-water solution with different Φ_{ethanol}. To investigate the influence of solvent temperature, water with temperature of 4ºC, 25ºC, and 35ºC were respectively used for helicoidal cisternae stacks formation. To trap fluorescent molecules in the cisternae stacks during the reorganization process, water solution containing 0.01 mg/mL Rhodamine B isothiocyanate-Dextran (~70 kDa) was used for helicoidal cisternae stacks formation. To investigate their morphology evolution, the helicoidal cisternae stacks were treated with heating or shaking in solution containing Rhodamine B isothiocyanate-Dextran (~70 kDa).

Molecules loading and enzyme catalytic reactions in the helicoidal cisternae stacks. For the efficient loading of molecules, helicoidal cisternae stacks composed of DPPC/DMPS/ Biotinyl Cap PE (*w*/*w*/*w*, 7/2/1) were formed through the reorganization of stacked bicelles. Rhodamine B isothiocyanate-Dextran molecules were encapsulated by incubating the cisternae stacks containing Biotinyl Cap PE with different concentrations of avidin for 2h. To control the reversible compression and expansion of the cisternae stacks encapsulated with fluorescent molecules, the cisternae stacks were reversibly dispersed in PBS for compression and water for expansion. A microreactor based on the 'breathing' helicoidal cisternae stacks was constructed by incubating the cisternae stacks in 0.4 U/mL HRP for enzyme encapsulation. The enzyme catalytic reactions were initiated by adding Amplex Red and H_2O_2 in the cisternae stacks dispersion to final concentrations of 100 μM and 40 μM, respectively. The enzyme catalytic reactions in both compressed cisternae stacks and expanded cisternae stacks were performed and compared.

Characterization. Fluorescence images were taken under a fluorescence microscope (Olympus IX73, Japan) and a confocal laser scanning microscope (Olympus FV 3000, Japan). To obtain the 3D morphologies of the helicoidal cisternae stacks, the cisternae stacks were visualized in serial sections, each 0.32 μm thick, by confocal laser scanning microscope. To obtain the 3D morphologies of the helicoidal cisternae stacks formed in NaCl solution, $CaCl_2$ solution, and low pH solution, after the incubation in the above solvent media for 4h, the cisternae stacks were dispersed in water to form loosely packed structures for the better characterization by the confocal laser scanning microscope. The images in Z-stacks were aligned and reconstructed using the software Matlab 2012. DSC experiments were carried out using a Mettler-TOLED DSC3 differential scanning calorimeter. The heating of the stacked bicelles samples was performed in the temperature range 30~50 °C at the scan rate of 1°C/min. To obtain the small angle X-ray scattering measurement result, the stacked bicelles samples were firstly concentrated to ~10 mg/mL via centrifugation. The measurement was performed at 25ºC by a SAXSpace small angle X-ray scattering instrument (Anton Paar, Austria, Cu-Kα, $\lambda = 0.154$ nm).
